# The lifesaving effects of cardiac adhesions

**DOI:** 10.1016/j.radcr.2023.04.018

**Published:** 2023-05-12

**Authors:** Adam L. Richardson, Olivia K. Richardson, Rebecca L. Guan, Ryan J. Anderson, Tracy L. Van Meter

**Affiliations:** a Department of Radiology, University of Louisville School of Medicine, 530 S Jackson St, Louisville, KY 40202, USA; b Department of Radiology and Medical Imaging, University of Virginia Health System, Charlottesville, VA, USA

**Keywords:** Cardiothoracic imaging, Cardiac surgery, Diagnostic radiology, Emergency radiology, Pericardial adhesions, Ventricular pseudoaneurysm

## Abstract

Patients that incur myocardial disruption from penetrating cardiac injuries have an average 6%-10% expectancy rate of reaching the hospital alive. If prompt recognition on arrival is not immediate, the morbidity and mortality are significantly higher due to the secondary physiologic sequalae of either cardiogenic or hemorrhagic shock. Even after a triumphant arrival at a medical facility, out of that 6%-10%, half of those patients are not expected to survive. The unique significance of the presenting case breaks this tradition, expanding past the paradigms and issuing an exceptional understanding of the protective effects that cardiac surgery can futuristically cause through preformed adhesions. In our case, the cardiac adhesions achieved this by containing a penetrating cardiac injury that had caused complete ventricular disruption.

## Introduction

The primary limitation of any penetrating cardiac injury is the timely identification of the diagnosis and providing proper treatment for the sustainment of life. It is essential to point out that in terms of physiopathology, once the physiological delivery of oxygen is interrupted by a penetrating injury, the heart attempts to adjust to meet the metabolic demands of the body as cardiac tamponade, exsanguination, conduction abnormalities, and mechanical failure are all possible consequences of penetrating cardiac injuries. Each of these can potentially cause damaging implications, but all eventually bring about a similar loss in cardiac output, triggering compensatory mechanisms to take effect. If these compensatory strategies cannot adequately overcome the inadequacies of the heart and maintain a certain degree of centralized homeostasis to the vital structures, then further and often rapid deterioration ensues, leading to the general statistic that 90% of these victims expire before reaching the hospital. Of those that do reach the hospital, 50% of the risk of further mortality after arrival [1,2]. This is why the “father of physiology,” Hermann Boerhaaeve, labeled penetrating cardiac injuries as fatal, irrespective of circumstances [3].

This report will discuss a case that explores cardiac surgery's unique and later effects as a patient defied the odds of a penetrating cardiac injury. The case is not only educational but provides further proof of radiology's indispensability in penetrating thoracic injuries.

## History of presentation

A 69-year-old Caucasian female with a history of chronic obstructive pulmonary disease, coronary artery disease status post-triple coronary artery bypass grafting (CABG) in 2017, and heart failure with preserved ejection fraction arrived at the hospital by emergency medical services (EMS) after multiple stab wounds were inflicted from an unknown assailant while she was sleeping. On the primary survey, the patient had multiple penetrating wounds to her upper body: one at the mid-left anterior neck located within zone 2; two at the left anterior chest wall within the region of the “cardiac box”; singular wounds to the epigastrium and right upper quadrant; and multiple superficial lacerations to the face and scalp. The patient was negative for hypotension, jugular venous distention, and muffled heart sounds, suggesting the absence of a pericardial effusion, through the physical exam findings that are classically known as Beck's triad. Inspection of the neck showed a clear violation of the platysma, with some questionable effervesce of sanguineous fluid at the site. On arrival, the patient appeared anxious; however, there were no concerns about airway compromise. She was hemodynamically stable, and the extended focused assessment with sonography in trauma (E-FAST) was negative upon initial evaluation in the trauma bay. However, as imagined, the exam was limited and partially compromised due to the overlying subcutaneous emphysema from the penetrating wound. Chest radiograph was unremarkable for mediastinal enlargement, cardiac enlargement or contour disturbances, layering effusions, or lucencies to indicate intra-thoracic or mediastinal air that would further point to an acute cardiopulmonary pathology. Given the hemodynamic status, negative thoracic findings, and the warranted concerns for the incisional neck wound and peritoneal violation, a decision was made to proceed to the operating room for further exploration.

**Fig. 1:**
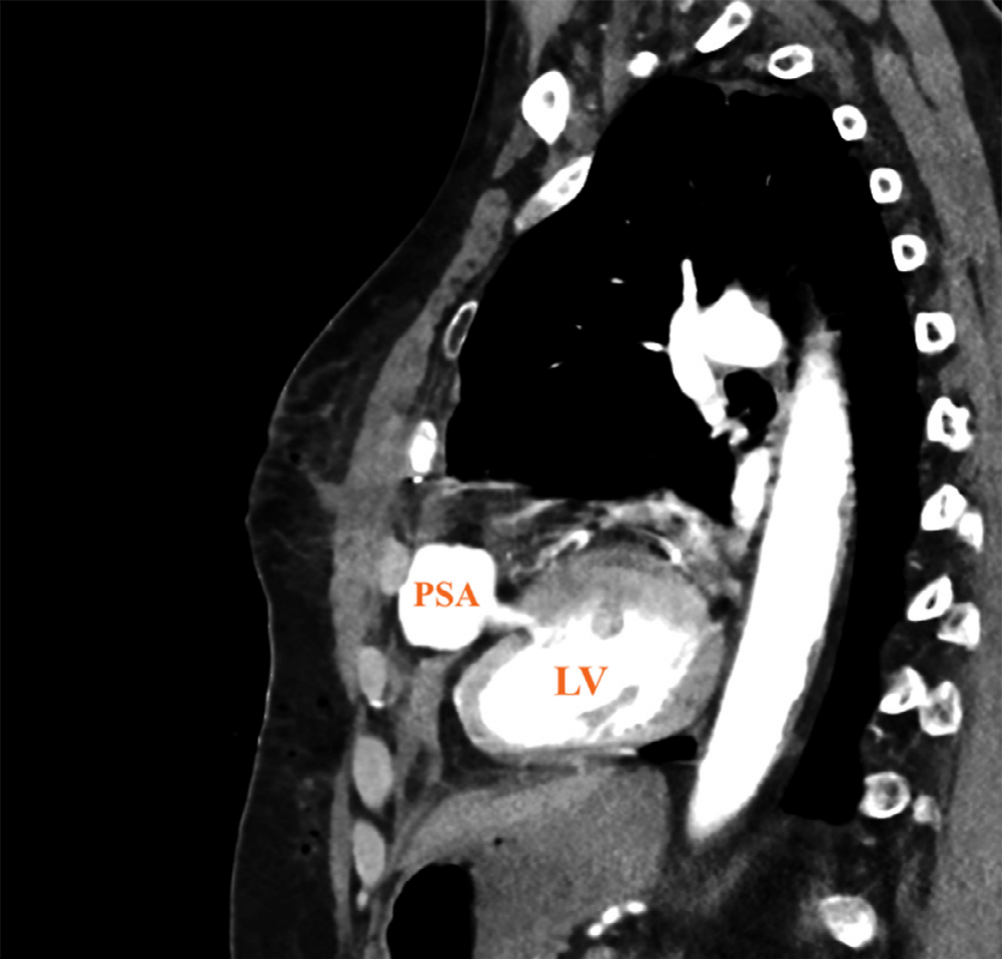
Sagittal Chest CT emphasizing the penetrating trajectory of the knife, coursing posteriorly above the left fifth rib and through the anterior wall of the left ventricle (LV). The penetrating wound traveling from the anterior chest wall to the left ventricle measured 10 cm in length. The large ventricular pseudoaneurysm (PSA) communicates with the left ventricle (LV).

**Fig. 2:**
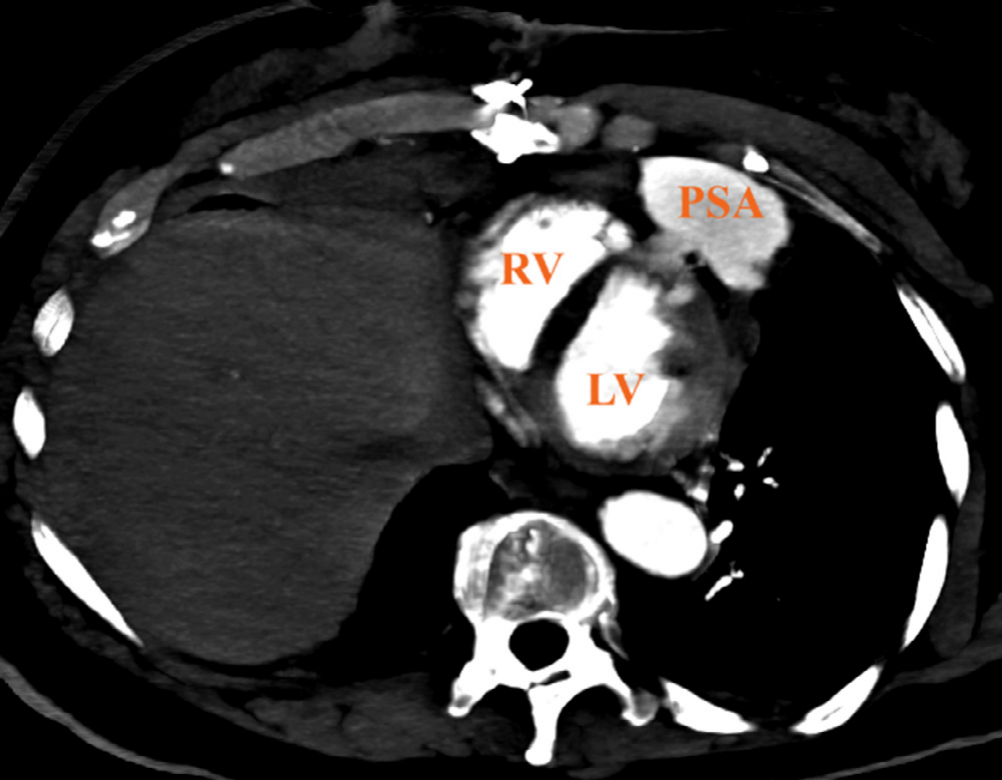
Axial MIP Chest CT identifying the communicating defect of the contained pseudoaneurysm (PSA) involving the right ventricle (RV) and left ventricle (LV). The violation to the right ventricle measured roughly 0.7 × 0.5 × 0.2 cm and an estimated 0.7 × 1.1 × 0.2 cm defect at the left wall. The body of the pseudoaneurysm measured 6.7 × 3.1 × 3.5 cm.

The patient underwent an exploratory laparotomy, hepatorrhaphy, neck exploration, and closure of her incurred lacerations in the operating room. As mentioned, during surgery, the team identified and repaired the liver and splenic lacerations; otherwise, enteric and diaphragmatic structures were intact on inspection. Postoperatively, a chest computed tomography (CT) with intravenous contrast was obtained to investigate the penetrating thoracic injuries, which unsuspectingly revealed a violation of the anterior cardiac wall. Contrast extravasation had pooled into a loculated area superior and anterior to the left ventricle, stemming from the anterior margin of the interventricular septum (Fig. 1, Fig. 2). At the time of the exam, when considering the presence of contrast material communicating through the bilateral ventricles with a clear tract of trajectory from the penetrating knife, concerns were raised about a contained rupture. In a self-limiting fashion, multiple other stab wounds were found at the left pectoralis major muscle, forming a small but appreciable hematoma. In addition, multiple scattered foci of soft tissue gas were within the anterior chest and abdominal walls.

## Clinical decision

**Fig. 3:**
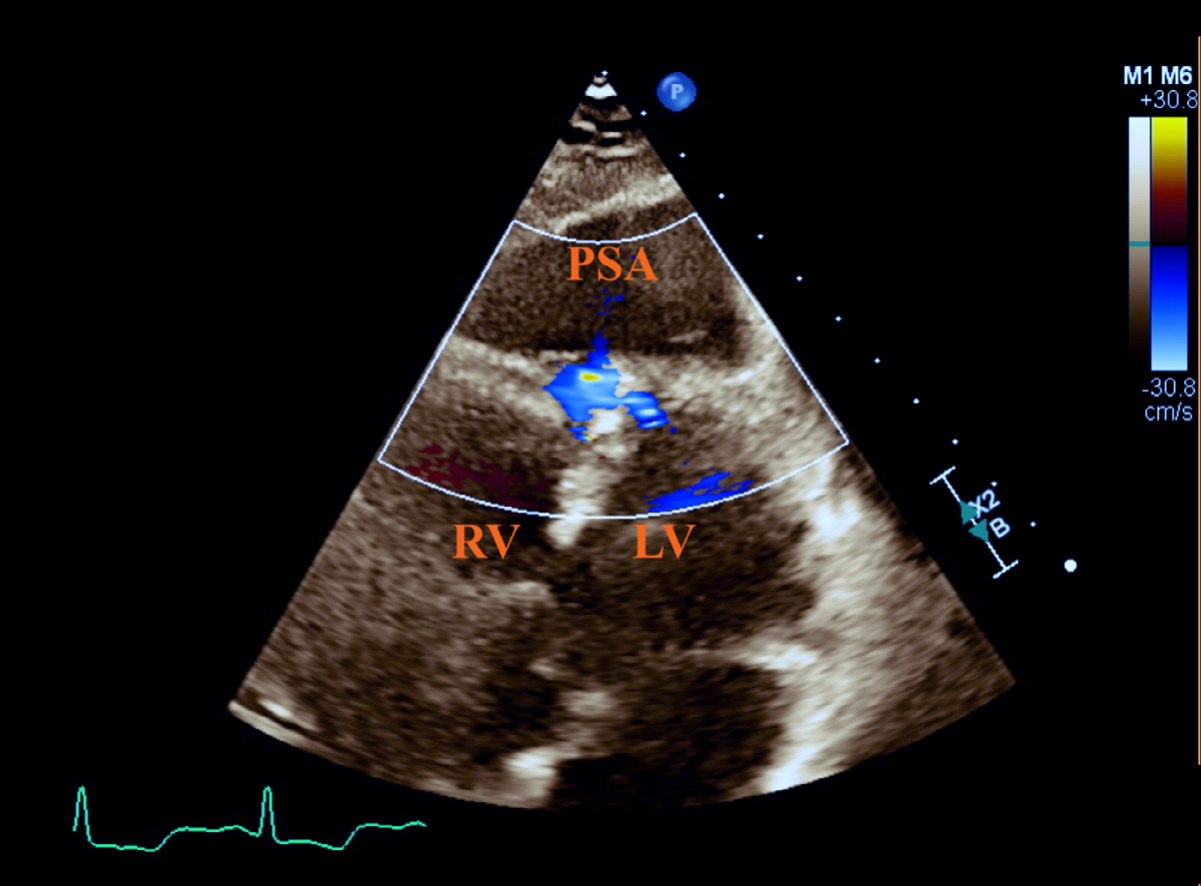
Transthoracic apical 4-chamber echocardiogram with color Doppler flow across the anterior right and left ventricular walls, centering over the interventricular septum and emptying into the ventricular pseudoaneurysm (PSA).

A STAT transthoracic echocardiogram (TTE) was ordered following CT imaging to confirm and further evaluate the findings. Once performed, the TTE confirmed the ventricular pseudoaneurysm from CT, demonstrating the disruption of cardiac myocardium at the apex of the right and left ventricular walls (Fig. 3).

Cardiothoracic surgery proceeded with presurgical utilization of a STAT cardiac catheterization to aid in the diagnostic value and planning of the operation in hopes of optimizing outcomes with a final surgical repair. The resulting coronary angiogram found no identifiable traumatic injuries to the coronary arteries, so cardiothoracic surgery moved forward with the redo median sternotomy and cardiac repair.

Once the redo median sternotomy was underway and the sternum crossed, cardiopulmonary bypass was initiated. The adhesions were dissected over the left mediastinal and thoracic pleura first. The remaining adhesions were subsequently lysed for further inspection of the right and left ventricles, later revealing the laceration paralleling and abutting the left anterior descending coronary artery (LAD). The ventricular rupture was repaired by utilizing horizontal mattress sutures, with portions of the suture needing to be burrowed beneath the undersurface of the LAD as it passed through the junctional border of the apical anterior and septal portions of the left ventricle. A bridge between the 3 components of the right and left ventricular walls and the interventricular septum through approximation at certain portions of the repair. After completion of the repair, the repair was investigated through an intraoperative ultrasound to ensure an efficacious repair. Afterward, cardiopulmonary bypass was taken off gradually without difficulties, and with no evidence of bleeding, Bioglue was applied directly over the repair. The thoracic cavity was then closed with thoracic and mediastinal drains in place.

One year later, the patient has fully recovered and is back to her active baseline before the event. Like before the accident, she suffers from mild shortness of breath while climbing stairs but denies angina or chest pain upon exertion. In addition, she has no lower extremity edema, ascites, paroxysmal nocturnal dyspnea, or orthopnea. Her most recent echocardiogram revealed a 50%-55% ejection fraction, coinciding with her pre-traumatic diagnosis of heart failure with preserved ejection fraction.

## Discussion

Penetrating cardiac injuries most commonly include the right ventricle, often because of its anterior location. A singular injury to the right ventricle can decrease cardiac output by as much as 40% due to impaired contractility and the downstream effects of diminished preload. Moreover, the mortality rate only increases with further chamber involvement. When evaluating a subset of patients that survived long enough for an operational repair with 2-chamber involvement, the evidence suggests that penetrating injuries involving the right and left ventricles, similar to our case, cause the second-highest mortality rate of all penetrating cardiac injuries, behind only left-sided atrium and ventricle injuries. Based on the statistical analysis of 122 cases, patients take on an 84% mortality rate during the repair when a right and left ventricle injury occurs [4]. In our case, the patient was beyond fortunate that her pre-formed adhesions contained the rupture and prevented the rapid exsanguination of blood from enveloping the thoracic cavity.

The burden of cardiac adhesions forming after cardiac surgery is an expected phenomenon. However, the negative consequences of adhesions are only sometimes apparent once performing a redo median sternotomy, which accounts for up to 10% of annual cardiac surgeries. The adhesions are densely formed to and around the cardiac and mediastinal structures, altering the facial planes and distorting the typical anatomic structures, which not only increases operative times but also has the potential to increase complications if not adequately accounted for during the procedure [5]. While detrimental during redo procedures, they proved beneficial in our particular case by providing the scaffolding framework that allowed for the proper containment and formation of a pseudoaneurysm.

Ventricular pseudoaneurysms are understood to form due to an insult causing myocardial necrosis, commonly occurring after myocardial infarctions, surgery, infection, and trauma. Once necrosis ensues, the myocardial tissue integrity is lost. If it cannot meet the demands of the usual wall stresses, cardiac rupture will follow, resulting in cardiac tamponade and death. However, less commonly, if the overlying pericardium or scar tissue contains the rupture, this will form a ventricular pseudoaneurysm. Ventricular pseudoaneurysm formation occurs so infrequently that it has a 0.26% incidence rate [6]. Noteworthy enough, a case of this type has yet been described in the literature, where containment of an acute penetrating injury led to the immediate formation of a ventricular pseudoaneurysm after pre-formed cardiac adhesions contained the free ventricular wall rupture. While fortuitous in its occurrence, the significance of this documented case builds on to the conceptual etiologic occurrences of ventricular pseudoaneurysm formation and the remarkable case of a patient benefitting from cardiac adhesions.

## Conclusion

In a scenario where seconds can be the difference between life and death, a patient diagnosed with cardiac disruption is unlikely to survive. In our case, the patient survived against the odds due to the added protective and unintended benefit from her original cardiac surgery many years prior. The intended purpose of this case is to highlight a new physiopathology and raise awareness of the unique protective effects of pericardial adhesions in saving a patient's life.

## Patient consent

The patient has provided his consent, confirming that written, informed consent has been granted to publish their case.
